# Intentions and barriers to help-seeking in adolescents and young adults differing in depression severity: cross-sectional results from a school-based mental health project

**DOI:** 10.1186/s13034-024-00775-3

**Published:** 2024-07-15

**Authors:** Sabrina Baldofski, Jelena Scheider, Elisabeth Kohls, Sarah-Lena Klemm, Julian Koenig, Stephanie Bauer, Markus Moessner, Michael Kaess, Heike Eschenbeck, Laya Lehner, Katja Becker, Jennifer Krämer, Silke Diestelkamp, Rainer Thomasius, Christine Rummel-Kluge, the ProHEAD Consortium

**Affiliations:** 1 Department of Psychiatry and Psychotherapy, Medical Faculty, Leipzig University, Leipzig, Germany; 2 Department of Psychiatry and Psychotherapy, University of Leipzig Medical Center, Leipzig University, Leipzig, Germany; 3 Department of Child and Adolescent Psychiatry, Psychosomatics and Psychotherapy, Faculty of Medicine and University Hospital Cologne, University of Cologne, Cologne, Germany; 4 Centre for Psychosocial Medicine, Center for Psychotherapy Research, University Hospital Heidelberg, Heidelberg, Germany; 5 German Center for Mental Health (DZPG), partner site Mannheim/Heidelberg/Ulm, Mannheim, Germany; 6 University Hospital of Child and Adolescent Psychiatry and Psychotherapy, University of Bern, Bern, Switzerland; 7 Department of Child and Adolescent Psychiatry, Centre for Psychosocial Medicine, University Hospital Heidelberg, Heidelberg, Germany; 8 Department of Psychology, University of Education Schwäbisch Gmünd, Schwäbisch Gmünd, Germany; 9 Department of Child and Adolescent Psychiatry, Psychosomatics and Psychotherapy, Medical Faculty of the Philipps-University Marburg, Marburg, Germany; 10 Center for Mind, Brain and Behavior (CMBB), University of Marburg and Justus Liebig University Giessen, Marburg, Germany; 11 German Center for Addiction Research in Childhood and Adolescence, University Hospital Hamburg-Eppendorf, Hamburg, Germany

**Keywords:** Help-seeking, Young adults, Adolescents, Barriers, Depression

## Abstract

**Background:**

Mental health problems, such as depression, have a high prevalence in young people. However, the majority of youths suffering from depression do not seek professional help. This study aimed to compare help-seeking behavior, intentions and perceived barriers between youthswith different levels of depressive symptoms.

**Methods:**

This cross-sectional study is part of a large-scale, multi-center project. Participants were *n* = 9509 youths who were recruited in German schools and completed a baseline screening questionnaire. Based on their depressive symptoms, youths were allocated to the following three subgroups: (a) without depressive symptoms, (b) with subclinical symptoms, (c) with clinical symptoms (measured by PHQ-A). Quantitative analyses compared previous help-seeking behavior, help-seeking intentions and perceived barriers (Barriers questionnaire) between these subgroups. An additional exploratory qualitative content analysis examined text answers on other perceived barriers to help-seeking.

**Results:**

Participants were mostly female (*n* = 5575, 58.6%) and 12 to 24 years old (*M* = 15.09, *SD* 2.37). Participants with different levels of depressive symptoms differed significantly in help-seeking behavior, intentions and perceived barriers. Specifically, participants with clinical depressive symptoms reported more previous help-seeking, but lower intentions to seek help compared to participants without symptoms (all *p* < 0.05). Participants with subclinical depressive symptoms reported a similar frequency of previous help-seeking, but higher intentions to seek help compared to participants without symptoms (all *p* < 0.05). Perception of barriers was different across subgroups: participants with clinical and subclinical depressive symptoms perceived the majority of barriers such as stigma, difficulties in accessibility, and family-related barriers as more relevant than participants without depressive symptoms. Across all subgroups, participants frequently mentioned intrapersonal reasons, a high need for autonomy, and a lack of mental health literacy as barriers to help-seeking.

**Conclusions:**

Youths with higher levels of depressive symptoms are more reluctant to seek professional help and perceive higher barriers. This underlines the need for effective and low-threshold interventions to tackle barriers, increase help-seeking, and lower depressive symptoms in adolescents and young adults differing in depression severity.

**Trial registration:**

DRKS00014685.

## Background

Adolescents and young adults are a vulnerable age group with up to 19% of adolescents from 12 to 17 years old in Germany suffering from mental health problems [1] and 11% of 12–17 year old Europeans experiencing suicidal ideation [2]. However, of all youths (i.e., adolescents and young adults) with mental health problems, around 70–80% do not seek professional help and thus, do not receive treatment [3–5].

Seeking help for mental health problems can be defined as the process of attempting to “obtain external assistance to deal with a mental health concern” [6]. The help-seeking process can be mapped to the theory of planned behavior with the stages of attitudes, intentions, and finally, behavior [7]. More specifically, the help-seeking process requires certain steps of symptom identification, intention forming, knowledge, and help-seeking. First, the person has to identify symptoms and perceive them as a mental health problem, second, the help-seeking person needs to form the intention to seek help; third, the help-seeking person needs to know where to seek help; and finally, the help-seeking person is open to communicate problems to the accessible source [7].

Even youths experiencing mental crises delay seeking help [8]. This hesitant behavior might be a result of lower intentions to seek help. Specifically, adolescents experiencing higher levels of depressive symptoms reported lower intentions for potential help-seeking than those without depressive symptoms [9–12].

Different factors may hinder those in need to actually seek professional help. Two recent systematic reviews categorized barriers and facilitators that adolescents perceive in seeking help for a potential emotional problem [13, 14]. On an individual level, a lack of mental health literacy and a high need for autonomy in coping with their problems may be reasons for low help-seeking intentions. Moreover, social factors such as fear of stigma were frequently reported barriers. Fear of lacking confidentiality and other obstructive perceptions of therapeutic relationships (e.g., fear of being judged or not taken seriously) may be other barriers to help-seeking in adolescents [13, 14]. Besides these attitudinal barriers, structural factors such as lack of time and resources may also impede help-seeking [10, 15]. Further, previous help-seeking behavior is seen as a potential predictor for future help-seeking intentions and help-seeking behavior, as it could diminish barriers like stigma, enhance knowledge about mental health symptoms and accessible sources of help [16].

In addition to the previously mentioned barriers, specific psychopathology may present another hindering factor towards seeking professional help. Specifically, experiencing clinical depressive symptoms could create distinct barriers. Depressive symptoms such as feelings of worthlessness, guilt, and hopelessness as well as a lack in energy [17] are likely to hinder individuals from perceiving themselves as being worthy of help, having hope in getting an adequate treatment and getting better, and having the energy to search for help [9]. While potentially hindering factors increase with higher depressive symptomatology, perception for a need for help also increases [18].

To date, few studies examined relevant barriers towards help-seeking in youths with severe mental health conditions such as depression [9, 13, 19, 20]. For adolescents experiencing depressive symptoms, lack of trust, stigma, and shame were shown to be barriers to help-seeking [19]. Moreover, similar to adolescents without depressive symptoms, adolescents with depressive symptoms reported self-reliance or a high need for autonomy as reasons for low intentions to seek help [14]. Further, first evidence suggests that some barriers, such as fear of stigma, may be more prevalent in students suffering from depression and suicidal ideation than in students without these conditions [10]. Nevertheless, this result is based on a limited sample of over 18 year old college students [10]. It remains unclear whether the presence of depressive symptoms is associated with different perceived barriers to help-seeking in comparison to adolescents without this symptomatology. To our knowledge, no other study systematically compared help-seeking intentions and perceived barriers to help-seeking between adolescents with different levels of depressive symptoms.

The present study aimed to close this gap by comparing youthswith different levels of depressive symptoms regarding different aspects of help-seeking in a cross-sectional study. The study is based on a nationwide German multi-center school-based project [21]. Help-seeking intentions and barriers were examined across three subgroups of adolescents without depressive symptoms, with subclinical depressive symptoms, and with clinical depressive symptoms, respectively. The goals of this study were to compare these subgroups regarding (1) previous help-seeking behavior, (2) intentions to seek help for a potential mental health problem, and (3) perception of barriers to professional help-seeking. Additionally, possible effects of previous help-seeking on perception of barriers were explored. Quantitative methods as well as an additional exploratory qualitative content analysis were used to examine barriers to professional help-seeking. This study aimed to extend previous literature by differentiating between subgroups with different severity of depressive symptoms to examine help-seeking intentions and specific barriers. Further, this study differentiated between formal and informal help-seeking, thus enabling detailed insights into differences in help-seeking intentions and behaviour depending on the source of help.

Based on previous studies [22] and theory on the help-seeking process, it was hypothesized that the subgroup with clinical depressive symptoms shows higher previous help-seeking behavior compared to the other subgroups. In contrast, help-seeking intentions were expected to be negatively related to the different levels of depressive symptoms [10–12] with lower help-seeking intentions in the subgroup with clinical depressive symptoms compared to the other subgroups. With respect to the perception of barriers, no specific hypothesis was formulated as barriers in youths differing in severity of depressive symptoms have not been researched previously.

## Methods

### Participants and procedure

Recruitment took place between November 2018 and February 2022 within the research project “Promoting Help-seeking using E-technology for Adolescents” [21]. ProHEAD is a multi-center consortium and aims to improve help-seeking behavior in adolescents with clinical mental health symptoms, prevent mental disorders in adolescents with subclinical mental health symptoms, and strengthen their mental health. Participating students completed a baseline screening assessment, after which they were allocated to one of five online programs addressing mental health promotion, eating disorder symptoms, depressive symptoms, risky alcohol use, and promotion of help-seeking, respectively. Those RCTs as well as the study procedures, including details on recruitment, sampling, and all online programs, are described in-depth elsewhere [21, 23–27]. For the present sample, students were recruited in secondary schools in grades 6–13 as well as vocational schools located in five different regions of Germany (Hamburg, Heidelberg, Leipzig, Marburg, Schwäbisch Gmünd) and completed an online questionnaire.

This study analyzed data obtained through this initial baseline screening. Ethical approval was granted by the ethics committees of the leading study site, the Medical Faculty at the University of Heidelberg (Study ID: S-086/2018) and of each participating study center [21]. Before participation in the study, written informed consent was given by parents (or other custodian) and the participants. In participants 18 years and older, only the participants themselves had to provide their consent. All students ≥ 12 years of age with an informed consent were included in the study. *N* = 9954 students initiated the online screening. According to consortium agreement, participants who did not complete one of the questionnaires were excluded from the analyses (*n* = 445). This resulted in a final sample of *n* = 9509 students.

In the final sample, *n* = 5575 (58.6%) indicated their gender as female, while *n* = 3934 (41.4%) indicated their gender as male. Mean age was 15.09 years (*SD* 2.37), with a range from 12 to 24 years, while the majority of the sample (*n* = 8129, 85.5%) were minors. Family affluence was high for most participants (*n* = 6936, 72.9%) and the psychosocial risk in the majority of participants was low (no or low risk: *n* = 5056, 53.2%). Participants mostly attended schools for a university entrance (“Gymnasium”;* n* = 5042, 53.0%) or schools for all qualifications (“Gemeinschafts-, Ober- and Stadtteilschule”; *n* = 1660, 17.5%). In the total sample, participants showed an average PHQ-A score of *M* = 7.56 (*SD* 5.39), ranging from 0 to 27.

## Measures

### Sociodemographic variables

In the online questionnaire age, gender, migration background, family affluence, and family psychosocial risk factors were assessed. Migration background was operationalized through one question asking for parents’ country of birth. All participants with one parent or themselves being born outside of Germany were categorized as having a migration background. Family affluence was measured utilizing a German adaptation of the Family Affluence Scale [28]. The instrument consists of four items with different rating scales, asking for instance whether the participant has an own bedroom. Using the sum score of the four items, family affluence can be differentiated in low (0–2), medium (3–5), and high (6–9) family affluence [28]. Family psychosocial risk factors were measured using the Laucht-Index [29]. It consists of ten items that can be summarized to a sum score ranging from 0 to 10 with higher scores indicating higher psychosocial risk.

### Patient-health-questionnaire-9 for adolescents (PHQ-A)

The current level of depressive symptoms as well as suicidal ideation were assessed with the PHQ-A [30]. Nine items measured depressive symptoms. Utilizing a 4-point Likert scale ranging from 0 = “not at all” to 3 = “nearly every day”, those nine items assess the level of depressive symptoms within the last 14 days. A sum score of these items, reaching from 0 to 27, is computed, with higher scores indicating higher levels of depressive symptoms. Based on this score participants were categorized into three subgroups for the purpose of the present analysis: (a) participants without depressive symptoms (PHQ score between 0 and 9, Group 0); (b) participants with subclinical depressive symptoms (PHQ score between 10 and 14; Group 1); and (c) participants with clinical depressive symptoms (PHQ score between 15 and 27; Group 2). This categorization was based on previous studies using the PHQ-A for identifying different levels of depressive symptoms in adolescents [31, 32]. In addition, this categorization reflects the cut-off values used in the ProHEAD project to assign students with different symptomatology into the respective RCTs: students with a PHQ-score between 10 and 14 were categorized as being at risk for developing depression, i.e., experiencing subclinical depressive symptoms (and were thus referred to the respective RCT for prevention of depression), while students with a PHQ-score ≥ 15 were defined as having clinical depressive symptoms and were allocated to the RCT for students with severe psychopathology [21]. Further, students with a PHQ-score below 10 in the baseline screening questionnaire were allocated to the preventive RCT for students without mental health problems within the ProHEAD project [21]. To screen for suicidal ideation, two items asked respondents about their current suicidal thoughts within the past month as well as lifetime suicide attempts. Answer options were dichotomous (yes/no).

### Actual help seeking questionnaire (AHSQ)

Previous help-seeking behavior was assessed using the AHSQ [33]. Participants are asked to indicate if they had sought help for a mental health problem in the past. Answer options were categorical: 0 = “no”, 1 = “yes, during the last 12 months”, 2 = “yes, but more than 12 months ago”. If participants sought help in the past, they were then asked to report the source of help. Different sources were presented using 12 items, 11 of which each represented a different source of formal (e. g., school psychologist, teacher, psychiatrist) or informal help (e. g., friends, partner, parents). For the purpose of this study, the items were summarized in three different ways. First, a binary variable distinguished previous help-seeking or no previous help-seeking. Second, two binary variables categorized previous help-seeking from formal sources and from informal sources (participants answered to have sought help from any formal or informal source, respectively). In addition, a binary variable was computed to be used as a control variable and indicated formal or no formal previous help-seeking.

### General help seeking questionnaire (GHSQ)

The GHSQ [34, 35] was used to assess the intentions to seek help for hypothetical mental health problems from different sources. The instrument consists of 14 items, with 12 items identifying different formal (e.g., general practitioner, adolescent psychiatrist, psychotherapist) and informal (e.g., friends, parents) sources of help, one item being an additional free text field item and one item providing the option to indicate that one would not seek help at all. Participants indicated the likelihood of seeking help from different sources in the next four weeks if they were to suffer from mental health problems. Likelihood was rated on a 7-point scale, ranging from 0 = “extremely unlikely” to 7 = “extremely likely”. The authors of the GHSQ propose using three metric subscales ranging from 0 to 7 each, with higher scores indicating higher intentions for the respective behavior: formal help-seeking, informal help-seeking, and no help-seeking, with the latter being derived from the item “I would not seek help from anyone” [34].

### Barriers questionnaire

The validated “Barriers to Adolescents Seeking Help Scale” [36] was adapted and extended with additional items from the literature. While six items were derived from the original version of the BASH-B, six additional items on structural barriers, lack of mental health literacy, fear of being admitted to the (adolescent) psychiatric ward and on fear that others will worry were constructed based on an extensive literature search and the expertise of clinical psychologists and senior child and adolescent psychiatrists within the consortium. Participants were instructed to imagine suffering from mental health problems for a few weeks or months and were then asked if they would seek professional help in this case (i. e., from a psychiatrist or psychotherapist). If they indicated that they would *not* seek help, eleven items and one free text field item were used to evaluate potential reasons for not seeking professional help. Subjects rated to which degree each reason applied to them on a 4-point scale (1 = “does not apply” to 4 = “does apply”). To descriptively analyze frequencies of each item, items were binary coded into 1 = “barrier applies” (including answer options “does rather apply” and “does apply”) and 0 = “barrier does not apply” (including answer options “does rather not apply” and “does not apply”). In addition, following suggestions in previous studies in adolescents and young adults [36, 37], the items were summarized with respect to their content to six different categories (stigma, lack of mental health literacy, perceived family consequences, self-reliance and autonomy, difficulties in accessibility, fear of being admitted to a psychiatric ward). Cronbach’s Alpha for these categories was acceptable for stigma (α = 0.68), perceived family consequences (α = 0.60), and difficulties in accessibility (α = 0.67), only the category lack of mental health literacy showed low reliability (α = 0.17). Self-reliance and autonomy did consist of one item only. The free text field answers served as a basis for an additional exploratory qualitative content analysis to identify potential barriers that may not have been covered in the other items.

### Statistical analysis

Statistical analyses were performed using IBM SPSS Statistics version 27.0. Participants were allocated to one of three subgroups based on the PHQ-A (Group 0: without depressive symptoms; Group 1: with subclinical symptoms; Group 2: with clinical depressive symptoms) and were compared with respect to sociodemographic variables (age, gender, family affluence, psychosocial risk factors, migration background) and clinical characteristics (current suicidal ideation, lifetime suicide attempt; both based on PHQ-A). χ^2^-tests analyzed differences in categorical variables (gender, migration background, current suicidal ideation, lifetime suicide attempt) between the three subgroups. Due to non-normality of the data, continuous sociodemographic data (age, family affluence, psychosocial risk factors) were compared between subgroups using Kruskal–Wallis-*H* tests. In addition, intercorrelations between clinical study variables (subgroups based on PHQ-A, AHSQ, and GHSQ) were conducted and reported in the appendix.

To answer the first research question, previous help-seeking behavior (AHSQ) was compared between the three subgroups using χ^2^-tests. To answer the second research question, help-seeking intentions (GHSQ) were analyzed between the three subgroups. Three separate ANCOVAs compared the intentions to seek formal, informal, and no help (GHSQ), respectively. As significant differences in sociodemographic factors between the three subgroups with different levels of depressive symptoms appeared, age and family affluence were included as covariates in the ANCOVA, while gender represented another factor in the model instead of a covariate due to its categorical nature. Due to their significant intercorrelations with family affluence, psychosocial risk factors and migration background were considered redundant for further analyses and were not selected as control variables.

To answer the third research question, perceptions of different barriers (Barriers Questionnaire) were compared between the three subgroups (PHQ-A). Several separate ANCOVAs were conducted. To control for potential effects of previous help-seeking on perceived barriers, previous help-seeking from formal sources (help-seeking from formal sources vs. no/other help-seeking; AHSQ) was added as another factor. Similar to the previous analyses, other covariates in the ANCOVAs were gender (added as another independent variable), age and family affluence.

A two-tailed α = 0.05 was applied to statistical testing. For all analyses, post-hoc *t*- tests, post-hoc Dunn-Bonferroni tests and pairwise* z*-tests further compared subgroup differences for significant overall effects in ANCOVAs, in Kruskal–Wallis-*H* tests and in χ^2^-tests, respectively. For all ANCOVAs and post-hoc tests, Bonferroni correction was used to account for multiple testing. Here, *p*-values were corrected by the multiplication of the observed *p*-value by the number of tests [38]. Further, effect sizes were reported and interpreted: for ANCOVAs an η^2^ partial was used. An η^2^ partial = 0.01 was considered as a small effect, η^2^ partial = 0.06 as a medium effect and η^2^ partial = 0.14 as a large effect [39]. For χ^2^-tests as well as Kruskal–Wallis-*H* tests, Cramer’s V was used to estimate small (V = 0.1), medium (V = 0.3), and large (V = 0.5) effects [40].

Finally, free text field answers on additional barriers were analyzed qualitatively using a qualitative content analysis. MAXQDA qualitative software (version 22.1.1) served as a coding tool. Following Mayring’s inductive content analysis approach [41], categories that emerged during the coding process were added to a coding dictionary. Using the final coding dictionary, two authors coded all free text field answers independently. Raters coded each free text answer with one category. Inter-rater reliability was good with κ = 0.75 [42]. Frequencies of coding categories were analyzed descriptively in the three subgroups.

## Results

### Subgroup differences

In the sample, the majority (*n* = 6688; 70.3%) were classified as “without depressive symptoms” (Group 0), whereas *n* = 1706 (17.9%) were categorized into the second subgroup with subclinical depressive symptoms (Group 1). Finally, *n* = 1115 (11.7%) reported clinical depressive symptoms (Group 2). The three subgroups were compared with respect to sociodemographic characteristics (see Table 1). They differed significantly in gender (*p* < 0.001), with the percentage of female participants being significantly higher in the subgroup with clinical depressive symptoms (Group 2) compared to both other subgroups, and being higher in the subgroup with subclinical depressive symptoms (Group 1) compared to the subgroup without depressive symptoms (Group 0; all *p* < 0.001). Further, subgroups differed significantly in age (*p* < 0.001): Post-hoc tests revealed that only Group 0 was significantly younger than both other subgroups (*p* < 0.001), while the latter did not differ significantly (*p* = 0.999). A similar pattern appeared for migration background: Group 0 reported significantly less migration background than both other subgroups (*p* < 0.001), while the latter did not differ significantly (*p* = 0.999). With respect to socioeconomic status, the three subgroups differed in family affluence and in the Laucht-index for psychosocial risk (*p* < 0.001): Group 2 showed the lowest family affluence and highest psychosocial risk compared to both other groups, and Group 1 also showed a lower family affluence and higher psychosocial risk compared to Group 0 (all *p* < 0.001).

**Table 1:** Sample characteristics and comparison of subgroups based on depressive symptomatology (*N* = 9509)

	Group 0: without depressive symptoms (*n* = 6688)	Group 1: with subclinical depressive symptoms (*n* = 1706)	Group 2: with clinical depressive symptoms (*n* = 1115)	Test statistics	*p*	Effect size
Female gender,* n* (%)	3469 (51.9)^a^	1203 (70.5)^b^	903 (81.0)^c^	χ^2^ (2) = 455.16	< 0.001	*V* = 0.22
Age, *M* (*SD*)	14.90 (2.36)^a^	15.53 (2.35)^b^	15.55 (2.28)^b^	*H* (2) = 198.72	< 0.001	*r* = 0.11–0.22
Family affluence (FAS),* M* (*SD*)	6.69 (1.76)^a^	6.13 (1.89)^b^	5.93 (1.85)^c^	*H* (2) = 247.22	< 0.001	*r* = 0.06–0.15
Psychosocial risk factors, *M* (*SD*)	3.61 (1.15)^a^	4.09 (1.48)^b^	4.35 (1.60)^c^	*H* (2) = 210.056	< 0.001	*r* = 0.04–0.19
Migration background, *n* (%)	1823 (27.3)^a^	602 (35.3)^b^	415 (37.2)^b^	χ^*2*^ (2) = 74.44	< 0.001	*V* = 0.09
Current suicidal ideation (PHQ-A), *n* (%)	168 (2.5)^a^	229 (13.4)^b^	506 (45.4)^c^	χ^*2*^ (2) = 2080.83	< 0.001	*V* = 0.47
Lifetime suicide attempt (PHQ-A), *n* (%)	191 (2.9)^a^	175 (10.3)^b^	308 (27.6)^c^	χ^*2*^ (2) = 921.90	< 0.001	*V* = 0.31

Bonferroni correction applied

*FAS* family affluence scale, Psychosocial risk factors, Laucht-Index, *PHQ-A* patient-health-questionnaire-9 for Adolescents

Different superscript letters indicate significant subgroup differences in the respective variable

In the total sample, *n* = 903 (9.5%) reported suicidal ideation in the past month and *n* = 674 (7.1%) reported a past suicide attempt. These frequencies differed significantly between the three subgroups (all *p* < 0.001; see Table 1). Higher suicidal ideation and higher rates of previous suicide attempts were observed in Group 2 compared to both other subgroups, while Group 1 reported higher suicidal ideation and higher rates of previous suicide attempts than Group 0 (all *p* < 0.001).

Intercorrelations of clinical study variables are presented in Appendix Table 5. Depressive symptom grouping showed a significant negative correlation with previous help-seeking and a significant positive correlation with intentions to seek no help. Negative correlations between intentions to seek no help and intentions to seek formal or informal help occurred.

### Previous help-seeking behavior (AHSQ)

In total, *n* = 2843 (29.9%) participants reported to have sought help for mental health problems in the last year or before. Chi-square tests showed that the three subgroups differed significantly in the frequency of previous help-seeking (*p* < 0.001; see Table 2). Bonferroni corrected pairwise comparisons showed that Group 2 had highest rates of previous help-seeking, followed by Group 1, while Group 0 sought help least often in the past (all *p* < 0.001).

**Table 2:** Subgroup differences in previous help-seeking behavior and help-seeking intentions (*N* = 9509)

	Group 0: without depressive symptoms (*n* = 6688)	Group 1: with subclinical depressive symptoms (*n* = 1706)	Group 2: with clinical depressive symptoms (*n* = 1115)	Test statistics	*p*	Effect size
Previous help-seeking (AHSQ), *n* (%)	1416 (21.17)^a^	747 (43.79)^b^	680 (60.99)^c^	χ^*2*^ (2) = 1054.07	0.04	*V* = 0.31
Previous formal help-seeking, *n* (%)	502 (7.51)^a^	299 (17.53)^a^	317 (28.43)^b^	χ^*2*^ (2) = 24.21	< 0.001	*V* = 0.09
Previous informal help-seeking, *n* (%)	1357 (20.29)^a^	703 (41.21)^a,b^	635 (56.95)^b^	χ^*2*^ (2) = 6.55	0.04	*V* = 0.05
Help-seeking intentions (GHSQ)						
Intentions to seek formal help, *M* (*SD*)	2.56 (1.37)^a^	2.28 (1.23)^b^	2.33 (1.21)^b^	*F*(2, 9501) = 19.03	< 0.001	*η*^2^ partial = 0.00
Intentions to seek informal help, *M* (*SD*)	4.62 (1.38)^a^	3.91 (1.38)^b^	3.33 (1.31)^c^	*F*(2, 9501) = 331.11	< 0.001	*η*^2^ partial = 0.07
Intentions to seek no help, *M* (*SD*)	2.45 (1.73)^a^	3.26 (1.88)^b^	3.88 (1.91)^c^	*F*(2, 9501) = 225.70	< 0.001	*η*^2^ partial = 0.05

Controlled for age, family affluence, and gender. Bonferroni correction applied

*AHSQ* actual help seeking questionnaire, *GHSQ* general help seeking questionnaire

Different superscript letters indicate significant subgroup differences in the respective variable

With respect to previous informal help-seeking, the three subgroups differed significantly (*p* < 0.001): Groups 1 and 2 reported less often previous informal help-seeking compared to participants in Group 0 (both *p* < 0.001). No significant difference in informal help-seeking between Groups 1 and 2 emerged (*p* > 0.05).

Regarding previous formal help-seeking, the three subgroups also differed significantly (*p* < 0.001). Group 2 more often sought help from formal sources than Group 0 (*p* < 0.001), while Group 1 did not differ significantly from both other subgroups (*p* > 0.05).

### Help-seeking intentions (GHSQ)

An ANCOVA controlling for age, family affluence, and gender showed significant differences between all three subgroups in their intentions to seek formal, informal, and no help (all *p* < 0.001; see Table 2). Group 2 reported higher intentions to seek no help compared to Group 1 (all *p* < 0.001), while Group 0 reported lowest intentions to seek no help compared to both other groups (*p* < 0.001). Regarding intentions to seek informal and formal help, respectively, Group 2 reported lower intentions to seek informal help compared to Group 1 (*p* < 0.001), but in the intentions to seek formal help they did not differ from Group 1 (*p* > 0.05). Group 0 reported highest intentions to seek both formal and informal help (*p* < 0.001).

### Barriers to help-seeking

All participants who indicated in the barriers questionnaire that they would not seek professional help were analyzed regarding their perception of barriers. In total, *n* = 3220 (33.9% of the total sample) negated possible professional help-seeking.

#### Quantitative analysis of perceived barriers

Seven ANCOVAs analyzed perception of barriers depending on the three subgroups of depressive symptoms. For all barrier categories, a main effect for subgroup appeared while controlling for previous help-seeking from formal sources, sex, age and socioeconomic status (Table 3).

**Table 3:** Subgroup differences in perceived barriers to help-seeking (Barriers Questionnaire, *N* = 3220)

	Group 0: without depressive symptoms (*n* = 1847)	Group 1: with subclinical depressive symptoms (*n* = 781)	Group 2: with clinical depressive symptoms (*n* = 592)	Test statistic	*p*	Effect size
	*M* (*SD*)	*M* (*SD*)	*M* (*SD*)			*η*^2^ partial
Stigma	2.01 (0.93)^a^	2.42 (0.94)^b^	2.81 (0.97)^b^	*F*(2, 3206) = 9.91	< 0.001	0.01
Lack of mental health literacy	2.06 (0.79)^a^	2.27 (0.81)^a,b^	2.52 (0.75)^b^	*F*(2, 3206) = 7.40	0.001	0.01
Family-related barriers	2.06 (0.91)^a^	2.61 (0.90)^b^	3.04 (0.89)^c^	*F*(2, 3206) = 31.16	< 0.001	0.02
Self-reliance and autonomy	3.15 (0.53)^a^	2.97 (0.59)^b^	2.72 (0.56)^b^	*F*(2, 3206) = 15.06	< 0.001	0.01
Difficulties in accessibility	1.58 (0.66)^a^	1.77 (0.76)^b^	1.90 (0.82)^b^	*F*(2, 3206) = 6.04	0.002	0.00
Fear of being admitted to a (children and adolescent) psychiatric ward	2.26 (1.09)^a^	1.85 (1.09)^a,b^	2.34 (1.20)^b^	*F*(2, 3206) = 6.70	0.001	0.00

Reduced sample size due to nature of the questionnaire

Bonferroni correction applied. Controlled for age, family affluence, gender, and previous help-seeking from formal sources

Different superscript letters indicate significant subgroup differences in the respective variable

Post-hoc tests showed that perception of some barriers like stigma, self-reliance and autonomy as well as perceived difficulties in accessibility did not differ between Groups 1 and 2 (all *p* > 0.05). Barriers related to stigma and difficulties in accessibility were rated lower by Group 0 compared to the other two subgroups (all *p* < 0.05). For self-reliance and autonomy, a contrary pattern appeared. Here, Group 0 rated this barrier as more prevalent compared to both other subgroups (all *p* < 0.05). Other barriers like lack of mental health literacy and fear of being admitted to the (adolescent) psychiatric ward showed no difference between Group 1 compared to both other groups (*p* > 0.05), but were rated more prevalent by the Group 2 compared to Group 0 (all *p* < 0.001).

However, some barriers were rated differently between the subgroups. Group 2 indicated barriers associated with family-related reasons as more prevalent compared to both other groups and Group 1 rated them as more prevalent than Group 0 (all *p* < 0.05).

With respect to previous help-seeking from formal sources, which was included as a covariate, no significant main effect on perception of barriers appeared, but significant interaction effects with level of depressive symptoms emerged for stigma, *F*_(2, 3206)_ = 6.76, *p* < 0.001, and for fear of being admitted to an (adolescent) psychiatric ward, *F*_(2, 3206)_ = 7.26, *p* = 0.001.

Regarding effects between subgroups only participants who did *not* seek help from formal sources in the past rated stigma and fear of being admitted to the (adolescent) psychiatric ward differently depending on the level of depressive symptoms: Group 2 rated these barriers lower than both other subgroups (all *p* < 0.001). Participants who *did* seek help from formal sources did not differ significantly in those ratings (all *p* < 0.001).

Regarding effects within subgroups, Group 2 rated stigma and fear of being admitted to the (adolescent) psychiatric ward as more prevalent when they had not sought help from formal sources compared to when they had (*p* < 0.01). For stigma this effect was inverse in Group 0: those without previous help-seeking from formal sources rated stigma as less prevalent compared to those with previous help-seeking (*p* = 0.037).

#### Exploratory qualitative content analysis of perceived barriers

Among all participants who indicated that they would not seek professional help in the Barriers Questionnaire, *n* = 398 answered a free-text field to report additional barriers not measured in the questionnaire. In an additional exploratory qualitative content analysis, those answers were coded into nine categories consisting of 22 sub codes. The main categories included barriers related to stigma, lack of mental health literacy, family, self-reliance and autonomy, intrapersonal reasons, fear of consequences, negative experiences with therapy and perceived difficulties in accessibility. Out of all answers, *n* = 60 (15.1%) answers were jokes, random comments, unclear answers or remarks about previous or current diagnoses/treatment and therefore not assignable to any barrier category.

Participants in Group 2 varied more in their answers than the other two subgroups (see Table 4). Most frequently mentioned main categories in Group 2 were barriers including intrapersonal reasons (*n* = 18, 16.2% of all answers in this group) and barriers indicating a lack of mental health literacy (*n* = 18, 16.2%). Another frequently reported main category in this subgroup were barriers related to the participant’s family (*n* = 13, 11.7%), with subcategories including e. g. the fear that the family would know about the psychotherapy and would react negatively (*n* = 7, 6.3%) or the fear that others might worry about oneself (*n* = 6, 5.4%). Moreover, *n* = 12 (10.8%) participants reported negative previous experiences with psychotherapy. Less often, participants in Group 2 described barriers depicting the need for self-reliance and autonomy (*n* = 11, 9.9%). Only *n* = 1 (1.0%) answer was coded as the subcategory ‘preference to talk to family or friends’. With respect to the subcategories of difficulties in accessibility, *n* = 3 (2.7%) participants mentioned difficulties to find a therapist, a barrier never reported by both other subgroups.

**Table 4:** Frequencies of perceived barriers to professional help-seeking (free text field answers in the Barriers Questionnaire, *n* = 398)

Categories and sub-categories, n (%)	Group 0: without depressive symptoms (*n* = 194)	Group 1: with subclinical depressive symptoms (*n* = 93)	Group 2: with clinical depressive symptoms (*n* = 111)
Stigma	3 (1.5)	3 (3.2)	7 (6.3)
Shame	1 (0.5)	2 (2.2)	0 (0.0)
Fearing reactions of others	2 (1.0)	1 (1.1)	7 (6.3)
Lack of mental health literacy	45 (23.2)	22 (23.7)	18 (16.2)
No perceived need for therapy or problems/relativization of problems	20 (10.3)	10 (10.8)	7 (6.3)
Negative expectancies about effectiveness and psychotherapist	8 (4.1)	6 (6.5)	6 (5.4)
Lack of knowledge	3 (1.5)	1 (1.1)	1 (0.9)
Lack of motivation	14 (7.2)	5 (5.4)	4 (3.6)
Family-related barriers	2 (1.0)	1 (1.1)	6 (5.4)
Fear of parental reaction	0 (0.0)	3 (3.2)	7 (6.3)
Fear that others might worry about oneself	2 (1.0)	1 (1.1)	6 (5.4)
Self-reliance and autonomy	69 (35.6)	21 (22.6)	11 (9.9)
Preference to handle problems alone	18 (9.3)	7 (7.5)	10 (9.0)
Preference to talk to family or friends	51 (26.3)	14 (15.1)	1 (0.9)
Intrapersonal reasons	25 (12.9)	23 (24.7)	18 (16.2)
Difficulties talking about problems	18 (9.3)	14 (15.1)	11 (9.9)
General mistrust in others	4 (2.1)	2 (2.2)	3 (2.7)
Low self-esteem	3 (1.5)	7 (7.5)	4 (3.6)
Fear of consequences	6 (3.1)	1 (1.1)	4 (3.6)
Fear of being admitted to the psychiatric ward	2 (1.0)	0 (0.0)	1 (0.9)
Fear of negative consequences (in general)	4 (2.1)	1 (1.1)	3 (2.7)
Negative experience with therapy	10 (5.2)	6 (6.5)	12 (10.8)
Perceived difficulties in accessibility	5 (2.6)	1 (1.1)	9 (8.1)
No parental approval	3 (1.5)	1 (1.1)	5 (4.5)
Financial reasons	2 (1.0)	0 (0.0)	0 (0.0)
Time-related reasons	0 (0.0)	0 (0.0)	1 (0.9)
Non assignable	19 (17.1)	12 (12.9)	29 (14.9)

With respect to participants in Group 1, the most frequent main categories were barriers related to intrapersonal reasons (*n* = 23, 24.7%), self-reliance and autonomy (*n* = 21, 22.6%), and lack of mental health literacy (*n* = 22, 23.7%). The most frequently reported subcategories were difficulties to talk about one’s own problems because of fear of opening up or not being able to express one’s feelings (*n* = 14, 15.1%; subcategories of intrapersonal reasons) and the preference to talk to family or friends (*n* = 14, 15.1%; subcategories of self-reliance and need for autonomy).

Participants in Group 0 reported similar barriers as Group 1: the most frequent main category were barriers related to self-reliance and autonomy (*n* = 69, 35.6%). Here, reported subcategories were the preference to talk to family or friends (*n* = 51, 26.3%) or to handle problems alone (*n* = 18, 9.3%). Another frequently reported main category were barriers related to a lack of mental health literacy (*n* = 45, 23.2%). For instance, *n* = 20 (10.3%) would not perceive a need for therapy or would think that the problems were not severe (enough) and *n* = 14 (7.2%) reported a lack of motivation. The third most frequently mentioned main category were intrapersonal reasons mentioned in *n* = 25 (12.9%) answers with difficulties talking about one’s problem as the most common subcategory (*n* = 18, 9.3%).

## Discussion

Using both quantitative and qualitative approaches, this study found that youths differing in depression severity vary in their help-seeking behavior, intentions to seek help, and their perceptions of barriers to seek professional help. With respect to the first research question, the results support the hypothesized higher previous help-seeking behavior of adolescents with clinical depressive symptoms compared to others. Specifically, participants with clinical depressive symptoms reported to have sought help more often in the past compared to those without depressive symptoms. Participants with subclinical depressive symptoms, however, previously did not seek help more often than participants without symptoms. With respect to the second research question, the findings also confirm the hypothesized link between help-seeking intentions and depressive symptoms. Participants with clinical depressive symptoms reported lower intentions to seek further help in the future compared to both other subgroups, and participants with subclinical depressive symptoms had lower intentions to seek further help in the future compared to those without symptoms.

The results further showed that those who were currently most in need for help due to high levels of depressive symptoms also reported to have sought more help in the past. This is especially relevant as those with higher depressive symptoms also reported more past suicide attempts than the other subgroups. However, an alarming finding was that the intentions for future help-seeking seem to be negatively associated with the current level of depressive symptoms, even though those with higher depressive symptoms also reported higher current suicidal ideation and therefore would be in urgent need for help.

Overall, the findings of this study are in line with other studies, which found that depressive symptoms are associated with lower help-seeking intentions [10–12]. This study expands those results by including a wider range in age (12–25 years) and differentiating three subgroups of different levels of depressive symptoms. The fact that across a wider age range youths with subclinical depressive symptoms report lower intentions to seek further help than those without depressive symptoms suggests that intentions to seek further help decrease with increasing depressive symptoms.

The results suggest that differential perceptions of barriers could explain those differences in help-seeking intentions. Quantitative analyses showed that youths with currently severe depressive symptoms and low intentions to seek professional help indicated for most barriers that they were affected by them more strongly compared to those without severe depressive symptoms. Interestingly, this difference also appeared in the exploratory qualitative content analyses. For youths with lower levels of depressive symptoms, more than half of the mentioned barriers could be categorized as a need for autonomy, in particular as a preference to talk to their family or friends, or as a lack of mental health literacy, such as the negation of their potential problems. In comparison, youths with clinical depressive symptoms did not often mention the preference to talk to their family and friends, but were more concerned about their family finding out about their problem and reacting negatively. In general, youths with clinical depressive symptoms reported a broader variance of barriers. These differences in the perception of barriers and intentions could be a direct result of the psychopathology [9, 18]. The feeling of hopelessness, a depressive symptom, for instance could diminish the confidence that a therapist could help, and the feeling of guilt may increase the perception that one could be a burden if talking to others about one’s problems. An alternative explanation for these findings could be that participants with higher levels of depressive symptoms rated barriers as more prevalent as it is not only a hypothetical case for them. More research is necessary to explore this relationship of psychopathology and perception of barriers.

Despite various differences, some barriers seem to be prevalent in all youths regardless of their current depressive symptoms. Consistent with previous studies [43], youths perceived attitudinal barriers as more prevalent than structural barriers. Furthermore, across all subgroups a lack of mental health literacy, intrapersonal reasons such as difficulties to talk about one’s problems and a need for self-reliance and autonomy, reflected by the preference to handle problems alone or with support from family or friends, can be important barriers to seek professional help.

Interestingly, previous professional help-seeking was only in some cases related to perception of barriers in quantitative analyses. Perceptions of stigma and fear of being admitted to the (adolescent) psychiatric ward diminished when youths affected by clinical depressive symptoms did not seek professional help previously. For those who were currently not reporting depressive symptoms on the other hand seeking help from a professional in the past might have enhanced the perception of stigma. A special link of treatment experience to stigma has also been found in previous research [44]. Further, especially participants with clinical depressive symptoms reported that negative previous experiences with therapy would hinder them from seeking help from a professional. Future longitudinal research which closely examines those associations and takes into account the valence of previous experience is essential to differentiate the results [44].

These results provide relevant implications for both research and practice. Most importantly, the findings highlight the importance to pay attention to youths who are experiencing depressive symptoms, but are reluctant to seek professional help, and to consider their social and individual barriers. This study further underlines the need for targeted interventions [12]. Programs should encourage help-seeking at different stages and in different ways. The differences in intentions and barriers between three levels of depressive symptoms found here implicate that prevention but also early and regular interventions, which consider help-seeking barriers in their design or encourage further help-seeking, are necessary.

First, prevention for youths currently not experiencing depressive symptoms can beforehand reduce barriers and enhance mental health literacy. This in turn could increase early and future help-seeking. Second, early interventions targeting youths with subclinical depressive symptoms may reach those who are beginning to experience depressive symptoms and who already perceive higher barriers. For instance, early interventions may address help-seeking by delivering knowledge about when to seek help for depression and how to talk about one’s feelings and problems. They could also prevent a worsening of symptoms. Third, interventions in youths with depressive symptoms can specifically address attitudinal barriers like stigma or the need to handle problems alone and negative treatment experiences. Especially for adolescents, it is important to note that the parents play an important role in seeking and getting access to professional mental health care. Interventions should therefore also focus on the parents’ role in the help-seeking process.

To consider many barriers and different target groups, online interventions can be useful. They can build a low-threshold first step for those who perceive high barriers like the fear talking to strangers. Furthermore, online interventions with self-management tools can address barriers like the need for self-reliance and autonomy. Especially for youths, prevention and intervention in an online sphere could therefore be attractive [45, 46].

Clinical practice should especially consider the role of family and friends in the youth help-seeking process. On the one hand, family members may be a first support for youths and may represent trusted persons where youths easily seek help [15]. Thereby, they can also promote further help-seeking as youths stated that they relied on their family’s opinion. This facilitating role of the family is especially present for those with lower or without depressive symptoms. For those with clinical depressive symptoms on the other hand, relation to the family may present a barrier to seek help. Many children indicated that they fear a negative reaction of their parents, that they will worry or that they would not allow getting help from a professional. Online and low threshold services could be a good first contact point for youths who fear consequences by their parents and would therefore not seek help at other help-services like a psychiatrist like for instance the German chat counseling service “krisenchat”, [47].

One of the main strengths of this study is that the data bases upon a large sample with a wide age range including adolescents 12 years of age and older, and young adult students (18–25 years). Moreover, validated, internationally used questionnaires measured depressive symptoms, previous help-seeking and help-seeking intentions. Qualitative analyses further add to the validity of the study. A first limitation of this study is a potential selection bias due to exclusion of incomplete questionnaires and due to the necessity of parents’ consent for minors, which was impossible to eliminate for a large school-based sample. Second, all data are based on self-report by youths and not on clinical diagnoses or actual behavior. Even though questions were adapted to youths, some participants might have misunderstood the instruction, might have lacked mental health literacy. For instance, they could have had difficulties remembering their past behavior or could have not known what a mental health problem comprises. However, there is evidence that already children at the age of six can report on their health [48] and that the PHQ-A is a valid self-report measurement to detect depression in youths [49]. Third, measuring intentions is only an approximation of actual help-seeking behavior [50], and a divergence of intentions and behavior is possible [44]. Nevertheless, only previously validated questionnaires have been used to measure intentions and previous behavior [33–35] and intentions are a potent predictor of future behavior [51]. Fourth, the barriers questionnaire was limited to only one help-seeking source, even though the other questionnaires (AHSQ, GHSQ) did include more different formal sources like therapists and teachers. Future studies could use different questionnaires and measure barriers to different help-seeking sources. Further research should also consider other potential mediators and moderators. First studies have already targeted this with respect to students or adults [10, 44], but still other potentially influencing factors like suicidality as well as comorbid psychiatric disorders have not been addressed yet in a younger sample.

## Conclusions

Overall, this study shows the high need for effective interventions in adolescents to promote help-seeking of those in need. Despite more help-seeking experience and need, adolescents and young adults with higher levels of depressive symptoms appear to be more reluctant to seek help than those without depressive symptoms. Perceptions of barriers hindering to seek help from a professional vary with the level of depressive symptoms. Clinical interventions such as online services must consider those barriers and different target groups. Future longitudinal studies on associations between help-seeking behavior, intentions, barriers and depressive symptoms are needed.

## Data Availability

The datasets used and/or analysed during the current study are available from the corresponding author on reasonable request.

## Appendix

See Table 5.

**Table 5:** Intercorrelations of study variables (*N* = 9509)

	(1)	(2)	(3)	(4)	(5)
PHQ-A subgroups (1)					
AHSQ: previous help-seeking (2)	− 0.314*				
GHSQ: intentions to seek formal help (3)	0.092*	− 0.009			
GHSQ: intentions to seek informal help (4)	− 0.078*	− 0.066*	0.328*		
GHSQ: intentions to seek no help (5)	0.276*	0.038	− 0.209*	− 0.375*	

**p* < 0.001

## Abbreviations

AHSQ: Actual help seeking questionnaire
GHSQ: General help seeking questionnaire
PHQ-A: Patient-health-questionnaire-9 for adolescents
